# Self-Regulated Selective Surface Coating Enables Confinement of Adherent Cells in Closed Microfluidic Arrays

**DOI:** 10.1021/acs.analchem.5c02622

**Published:** 2025-11-17

**Authors:** Anna Kaehr, Guillaume Aubry, Hang Lu

**Affiliations:** † School of Chemical & Biomolecular Engineering, 1372 Georgia Institute of Technology, 311 Ferst Drive NW, Atlanta, Georgia 30332, United States; ‡ Interdisciplinary Program in Bioengineering, Georgia Institute of Technology, 311 Ferst Drive NW, Atlanta, Georgia 30332, United States

## Abstract

Phenotypical screening assays involving adherent cell culture are essential for research in cell biology and toxicology as well as drug discovery. Such assays often involve hundreds of culture chambers and monitor cell responses for hours. Microfluidic arrays have been widely used for cell assays; however, creating dense arrays of adherent cells is challenging because adherent cells may migrate over time. To confine cells, existing techniques rely on actuators such as valves or surface micropatterning; yet, both present important drawbacks. The actuator-based systems often require complex fabrication and equipment for operation. Noncontact surface patterning techniques for closed microfluidic channels typically require expensive reagents (e.g., photosensitive ones) and precise alignment, whereas contact techniques to pattern open surfaces make sealing the device after patterning difficult and are susceptible to contamination. Here, we present an inexpensive, selective surface-coating method for micropatterning proteins to confine adherent cells in closed microfluidic devices. Using capillary valves, pressure-driven flow, and sequential reagent delivery, we can spatially and temporally expose the channels to cell adhesion molecules and blocking agents separately. We show a large window of operating pressures, up to 4 psi, ensuring robust selective patterning. In the patterned devices, we also demonstrate successful cell culture for over 20 h, with cells remaining spatially confined. This technique offers a cost-effective and scalable solution for creating microarrays of adherent cells without complex equipment or expensive reagents. Its simplicity and ability to pattern in closed microchannels may also benefit applications beyond cell-based assays such as biomolecule detection and other surface-based processes.

## Graphical abstract

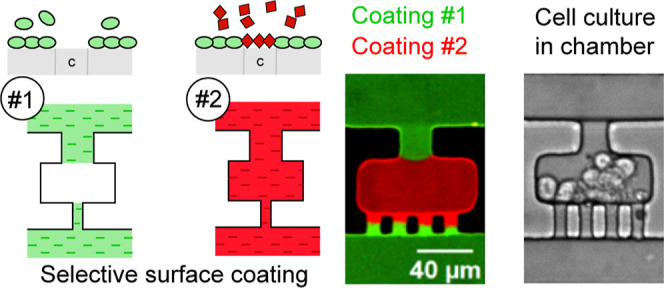

Adherent cell culture assays are used in a wide range of applications.^1,2^ Studying cell behavior over extended periods enables the determination of gene expression, signaling pathways, cell proliferation, and cell death in response to environmental perturbations.^3–5^ As the demand for high-throughput assays with reduced reagent consumption and advanced environmental perturbations grows, screening technologies are progressing beyond the traditional 96-well plate format, toward the development of smaller arrays with integrated flow control. However, doing so requires the arraying of cells into precise locations across hundreds of spots and ensuring that the cells remain in place throughout the assay, which presents a significant challenge. Since adherent cells are capable of migration, keeping them stationary for hours can be difficult.

Toward this goal, microfluidics has proven advantageous by enabling scaling down to the microscale and offering active controls over the cellular microenvironment^6^ but often at the cost of complexity. Many solutions exist for arraying cells over short periods of time.^7–9^ Hydrodynamic,^10–12^ acoustic,^13,14^ or electric force fields^15,16^ can efficiently direct cells or even single cells into arrays without the need for expensive equipment. Although initial trapping of cells in suspension is effective, the force fields may not be strong enough to retain adherent cells; stronger fields may be undesirable to avoid perturbing the cells. Other techniques use physical barriers to maintain the cells in place over longer periods of time. While effective, these techniques require actuators^17–20^ and multiphase systems^21,22^ that complicate the operation, or cause unwanted environmental perturbations.^23,24^ Finally, open surface systems that need an automated liquid handler to distribute cells or reagents^25,26^ show potential but are expensive.

Micropatterning is a promising technique to overcome this challenge as it allows for passively controlling cell behavior on surfaces.^27^ By coating only specific areas with cell adhesion molecules, cells are confined within these regions. Stamp-based methods, such as microcontact printing or microspotting,^28–32^ have demonstrated successful cell culture on open surfaces. However, adapting these approaches to pattern closed channels is difficult because physical contact is necessary, and therefore patterning must be performed before sealing the channels, which can lead to contamination, coating damage upon sealing, and misalignment of the channels with the pattern. For these reasons, performing surface coating inside closed channels is preferable. Another technique, photopatterning^33,34^ allows for surface patterning of bonded microfluidic devices but requires complex fabrication and expensive substrates (Supporting Information Table S1).

Additional micropatterning techniques exist that are attractive for their simplicity but are very limited in the type of patterns achievable (Supporting Information Table S1). Flow-based techniques, either using capillary forces or laminar flow, are simple as they rely on fluid transport in microchannels to deliver functional molecules.^35–37^ However, current capillary-flow-based methods use separate channels for supplying different patterning molecules, leading to continuous patterns along one dimension. Similarly, laminar flows generate continuous streams that return striped patterns. By design, neither method can produce separate spots. Therefore, there is a need for a method to create advanced patterns in closed microfluidic devices that follows a simple protocol and allows for feature registration at the microscale.

In this work, we present a simple and cost-effective method for cell confinement on a patterned surface, enabling both perfusion and precise spatial confinement of cells through targeted adhesion. The device fulfills two functions: it first distributes the cells across the array with a hydrodynamic-flow focusing approach,^10,23,38–40^ and second, retains the cells in the chambers longer-term using the selective surface coating. The core innovation lies in the flow-based surface coating method that creates complex patterns in closed microfluidic channels. By using capillary valves and different pressure commands to drive the liquids, we can first coat a blocking agent everywhere except the culture chambers and then cell adhesion molecules in target cell culture chambers. Performing the procedure is simple because the system relies only on applying sequential pressure commands, and the patterning is determined by the channel geometry without any need for alignment. Another benefit of this approach is scalability, which we demonstrate by selectively coating surfaces across hundreds of chambers organized in arrays. Finally, we demonstrate the potential for cell-based assays by successfully culturing adherent cells for over 20 h.

## Experimental Section

### Microfluidic Device Fabrication

The microfluidic devices were fabricated using conventional soft lithography techniques.^41^ The masters were obtained via successive photolithography steps with a negative photoresist (SU8, Kayaku AM) on silicon wafers. Three different masks were used to cast three layers of photoresist in order of increasing height: the back channel of the chamber, the front channel of the chamber, and the serpentine channel and chambers. The heights of the serpentine channel and chambers were kept at ∼40 μm across all masters. Multiple masters were created to vary the heights of the front and back channels, as indicated in the text.

The fabricated master was treated with tridecafluoro-1,1,2,2 tetrahydrooctyl-1-trichlorosilane vapor (Sigma-Aldrich) overnight to facilitate the release of poly­(dimethylsiloxane) (PDMS, Dow Corning SYLGARD 184) during the molding process. A mixture of 10:1 poly dimethylsiloxane (PDMS)/cross-linker was then poured on top of the wafer to obtain a thickness of ∼5 mm and cured in an oven at 70 °C overnight. Following this, PDMS slabs were cut for each device, access holes were punched with biopsy punches (1.5 mm diameter for the inlet, 1 mm for the outlet, Integra LifeSciences), and the PDMS devices were bonded to glass coverslips via plasma treatment using a plasma wand (Plasma Etch, Inc.): 30 s on a 30 × 40 mm glass coverslip and 30 s on a 20 × 20 mm piece of PDMS. To ensure that surface properties remain the same, one recommends keeping the surface clean at all times by covering the PDMS surface with a piece of tape until the bonding step and then storing the device in a box. Keeping the same time interval between plasma treatment and the coating procedure is good practice to build reproducibility from assay to assay. Note that some designs vary the number of back channels connecting a chamber to the serpentine but these variations do not impact the principle or the protocol of the selective surface coating method.

### Reagents

Bovine serum albumin (BSA), fibronectin (FN), and Pluronic F-127 were obtained from Thermofisher Scientific. For studying the robustness of the protocol, a food dye solution, 4% BSA solution, and 1% Pluronic solution were used. The food dye was dissolved in DI water. The BSA and Pluronic were dissolved in phosphate-buffered saline (PBS). For characterizing the protein patterning, BSA conjugated to fluorescein (1% BSA-FITC) was used for main channel blocking, and BSA conjugated to Texas Red (0.1% BSA-TXR) was used for chamber surface treatment. For cell adhesion studies, unconjugated BSA (4% solution) was used for main channel blocking, and FN (1 μg/mL solution) was used for chamber surface treatment. Protein solutions were prepared on the day of use by dissolving protein powder in PBS, centrifuging the solution at 4 °C, and filtering insoluble matter with a 0.20 μm syringe filter.

### Experimental Setup for Device Design Studies

DI water was used to displace air and fill the microfluidic device with liquid. Custom-made pressure boxes and MATLAB user interfaces (available upon request) were used to set command pressure to drive the liquids. Applied pressure was increased from 1 to 15 psi (or until complete filling of all capillary valves) in 0.25 psi increments with 10 s hold steps for system stabilization. The microfluidic device was imaged at 6.6× magnification on a dissecting scope (ZEISS Stemi SV11). Recordings were captured with a CCD camera (Lumenera, Infinity 2), and video analysis was performed in ImageJ. Note that the pressure box controlled via the MATLAB program was exclusively used for this set of experiments. In all other assays, the surface coating protocol was performed by hand-pushing a syringe.

### Device Characterization

To determine the “filling” regime of a given array, the water/liquid pressure was increased incrementally, and the status of each valve in the array was characterized. The “empty”, “filling”, or “filled” status was determined for each pressure command by assessing the fraction of valves in a particular regime and comparing that fraction to a threshold of 0.8. The “empty” regime goes from no pressure (an air-filled device) up to the pressure at which 80% of the valves still have no liquid entering the chambers. The “filling” regime is from the upper limit of the empty regime up to the pressure at which 80% of the valves still have some air in the chambers. The “filled” regime is defined as all pressures above the “filling” regime limit.

### Selective Surface Coating

Prior to use, dry devices were sterilized under UV light for 30 min and tubing and fittings were autoclaved. Blocking solution (4% BSA) was injected into the outlet of the device by gentle positive pressure (1 psi) via a pressure box. Pressure was applied until fluid filled the entire main channel and inlet of the device. The device was disconnected from the pressure source and not degassed, leaving air trapped in the chambers of the device. A 1.5 mm diameter pipet tip was inserted in the inlet and filled with 100 μL of blocking solution to compensate for evaporation. The device was incubated in a humidified box for 12 h at 4 °C for optimal blocking of the main channel.

Afterward, the blocking solution was removed from the reservoir and washed with PBS via gravity flow at ∼0.1 μL/min for 1 h. Fresh PBS was added to the reservoir, and the entire device was degassed with PBS to remove air from the chambers. Then, 100 μL of surface treatment solution (1 μg/mL FN) was added to the reservoir at the inlet and perfused via gravity flow. The device was incubated for 2 h at 25 °C for surface treatment of the unblocked chambers. Finally, the FN solution was removed from the reservoir, washed once with PBS, and replaced with 100 μL of fresh PBS prior to imaging.

### Cell Culture and Device Loading

The dimensions (width x length x height) of the devices used in the biological experiments (Figure 5) are (1) 20 μm × 30 μm × 20 μm for the inlets (front channel connecting serpentine to chamber); (2) 10 μm × 30 μm × 5 μm for the outlets (back channels connecting serpentine to chamber); (3) 20 μm × 50/70/90/110 μm × 20 μm for the different chambers; and (4) 40 μm × 40 μm for the serpentine. The adherent epithelial HT-1080 and fibroblast NIH3T3 cell lines (ATCC) were cultured in DMEM medium (ATCC) supplemented with 10% fetal BSA and 100 units/mL penicillin streptomycin (VWR). Cells were cultured in tissue culture flasks at 37 °C in a humidified 5% CO_2_ incubator. For device loading, cells were trypsinized with 0.25% trypsin–EDTA and resuspended as 0.5 × 10^6^ cells/mL solution. 50 μL of cell solution was pipetted into the inlet reservoir of the surface-treated device, and cells were loaded into the chambers by gravity flow (5 μL/h). Following cell loading, any remaining cell solution was removed from the reservoir and replaced with phenol-red free cell media (Thermofisher Scientific) for cell culture and subsequent imaging. The outlet tubing was placed lower than the device so that cell media perfused continuously through the device. Live–dead staining was performed the following morning with Calcein-AM and propidium iodide (Thermofisher Scientific).

### Cell Microscopy and Analysis

Confocal microscopy (Nikon W1 spinning disk confocal system) was performed to characterize the protein and cell patterning. Fluorescence profiles were determined by processing images in ImageJ. Cell patterning was also quantified in ImageJ by applying a binary mask of the main channel to the images of the array to differentiate cells in the main channel from cells in the chambers.

To establish the fraction of HT-1080 adherent cells after 20 h culture, the flow of cell culture medium was reversed in the devices. Nonadherent cells were pushed out of the chambers, while adherent cells stay. The fraction of adherent cells in the chambers was quantified as the fraction of cell remaining in the chamber after 1 min of reverse flow at 1 psi.

## Results and Discussion

### General Principle to Perform Selective Surface Coating in Closed Microfluidic Channels

Our objective of this work is to develop a simple, user-friendly selective surface coating technique to culture adherent cells in microfluidic arrays. To do so, one must create 2D patterns of adherent and blocking agent molecules (Figure 1a). This can be done by coating the chamber surfaces with adherent molecules to support the cell adherence and coating the rest of the device with blocking agent molecules to prevent the migration of cells outside the chambers. The difficulty resides in patterning closed microfluidic channels, where direct access to the surface is not possible.

**Figure 1:**
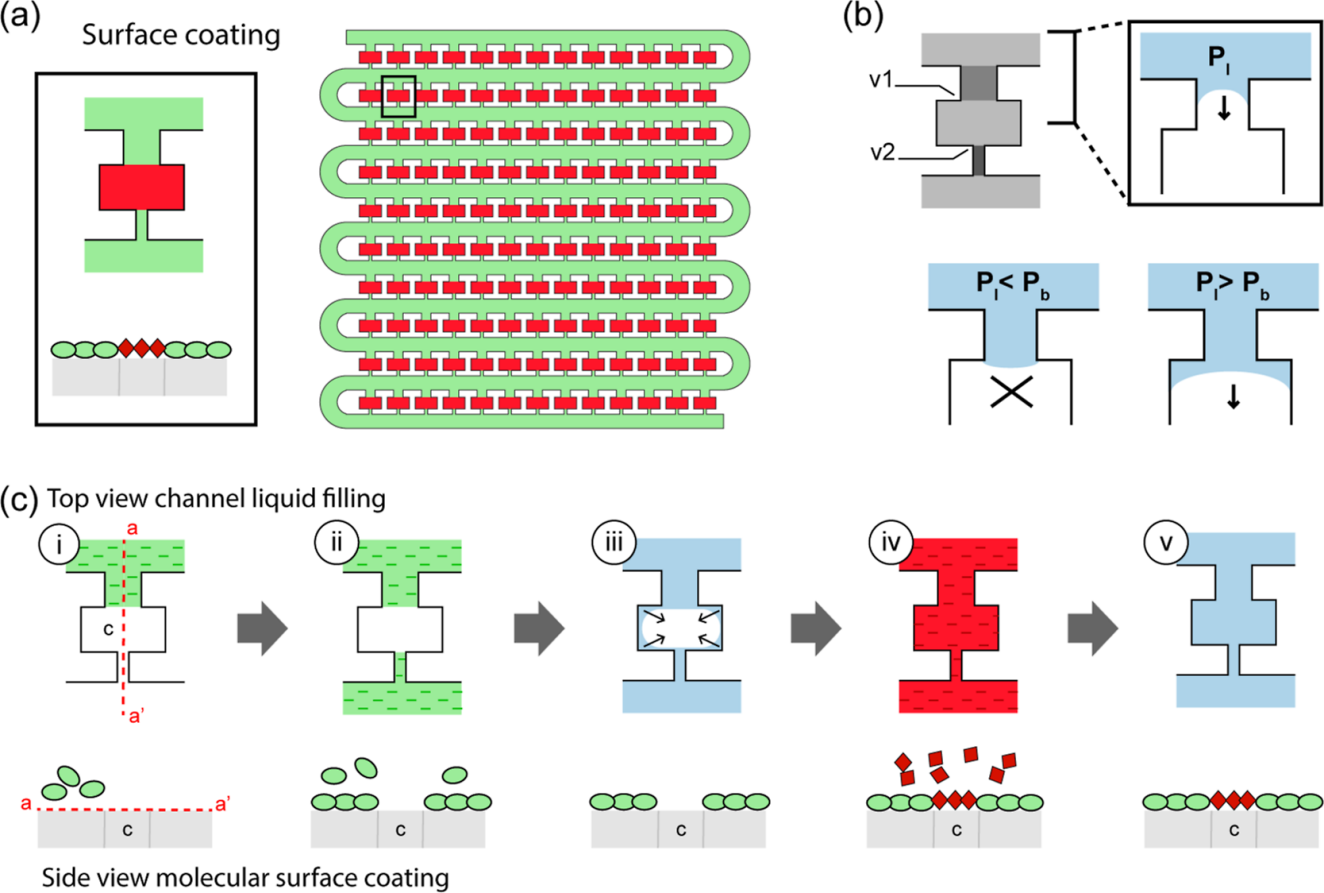
Selective surface coating in the closed environment. (a) 2D pattern in a cell trap array. (b) Layout of a single unit (in gray) showing the location of the two capillary valves v1 and v2; zoomed-in view of v1 regulating chamber filling as a function of the liquid pressure P_l_ and the valve burst pressure P_b_. (c) Sequence of liquid transport through the channels (top row) and the corresponding surface molecular state (bottom row). The dashed line (aa′) in (i) across the chamber indicates the location of the surface shown on the bottom row; “c” indicates the chamber location along the surface. (i) Introduction of blocking agent solution at P_l_ < P_b_. (ii) Incubation: the blocking agent coats all the channels except the chamber. (iii) Washing step and degassing (illustrated with arrows) at P_l_ > P_b_. (iv) Introduction of cell adhesion molecule solution and incubation. The cell adhesion molecules coat the available surface. (v) After a washing step, the surface is selectively coated with adhesion molecules in the chambers.

To overcome this challenge, we delivered the molecules in liquid and controlled the liquid delivery in selected areas of the device. The key mechanism is the use of capillary forces to stop pressure-driven flow in precise locations and, by applying different pressures, drive liquids to different areas of the device. The channel geometry plays an essential role in this strategy. The overall layout for arraying cells borrows from hydrodynamic flow-focusing arrays.^10,23,38–40^ The main structure is composed of a serpentine channel and chambers connected to the serpentine via a front channel and a back resistance channel (Figure 1b). Importantly, we designed both front and back channels so that their cross sections are smaller than the chamber’s cross-section. These features create capillary stop valves v1 and v2 (Figure 1b), characterized by their pressure burst P_b_, which allow control of liquid transport. For a positive pressure applied to the liquid in the channel (P_l_), the liquid fills in the serpentine channel and the front channel up to the location of the chamber. There, the local opening of the chamber generates curvatures of the liquid–air interface where interfacial tension balances the pressure difference in liquid and in air. At low pressures (P_l_ < P_b_), capillary forces pin the interface at the opening and the flow stops. When the liquid pressure is increased above the valve burst pressure, the interfacial tension is no longer strong enough to pin the liquid, and the liquid flows through the valve. Both valves v1 and v2 operated in a similar manner. We used them as gates to regulate liquid transport. As such, the geometry of v1 and v2 valves is essential to define precise wetting areas. However, to achieve selective coating, the sequence for delivering reagents and operating the capillary valves is equally important.

Figure 1c illustrates how we used the capillary valves for the selective surface coating for each chamber. In the first step, a blocking solution flows through the air-filled array at low pressure. The applied pressure P_l_ is lower than the valve’s burst pressure P_b_; therefore, the blocking agent fills the serpentine channel but not the chambers. The blocking agent will adsorb onto the channel’s surface when incubated for a certain length of time. After incubation and a washing step, buffer is introduced into the device using a pressure above the valve burst pressure. The air bubbles trapped in the chambers are either pushed out of the chambers and wash away via the main channel or defused through the PDMS around the chamber. Then, a solution of adhesion molecules is introduced to the chamber, and the adhesion molecules are allowed to adsorb onto the chamber surface. Finally, the device is patterned with cell adhesion molecules in the chamber and a blocking agent in the rest of the device, creating an effective 2D array pattern. This simple controlled transport of liquids dictates the patterning of the desired molecules on targeted regions of the device.

### Design of a Capillary Valve Network with the Flexible Operating Range

To create an array of hundreds of selectively coated regions using capillary flow requires precise, controlled fluid transport in two dimensions. One major challenge lies in designing capillary valves that can sustain the liquid driving pressure during the initial filling of the array. The driving pressure must be lower than the valve burst pressure and be high enough to fill the entire serpentine channel. Determining this driving pressure is not simple. As the liquid replaces air in the serpentine channel, the hydraulic resistance increases and the pressure drop across the liquid/air interface decreases. Therefore, for a fixed applied pressure during channel filling, upstream valves are exposed to a higher pressure than the downstream valves.

To establish the operating range for such a valve network, we studied the effect of the valve geometry on the performance of the array filling. We designed several chamber arrays with varying valve strength by adjusting the width and the height of the connecting channels (Figure 2a). For simplicity, we kept the front and back channels (valves v1 and v2) identical and studied a range of channel widths and heights that are relevant for mammalian cell assays. The front channels (v1) must be larger than the cells for loading the cells in the chamber, and back channels (v2) must be smaller than the cells for trapping the cells inside the chambers. We also fixed the main culture chamber widths to be at least twice the size of the valve widths.

**Figure 2:**
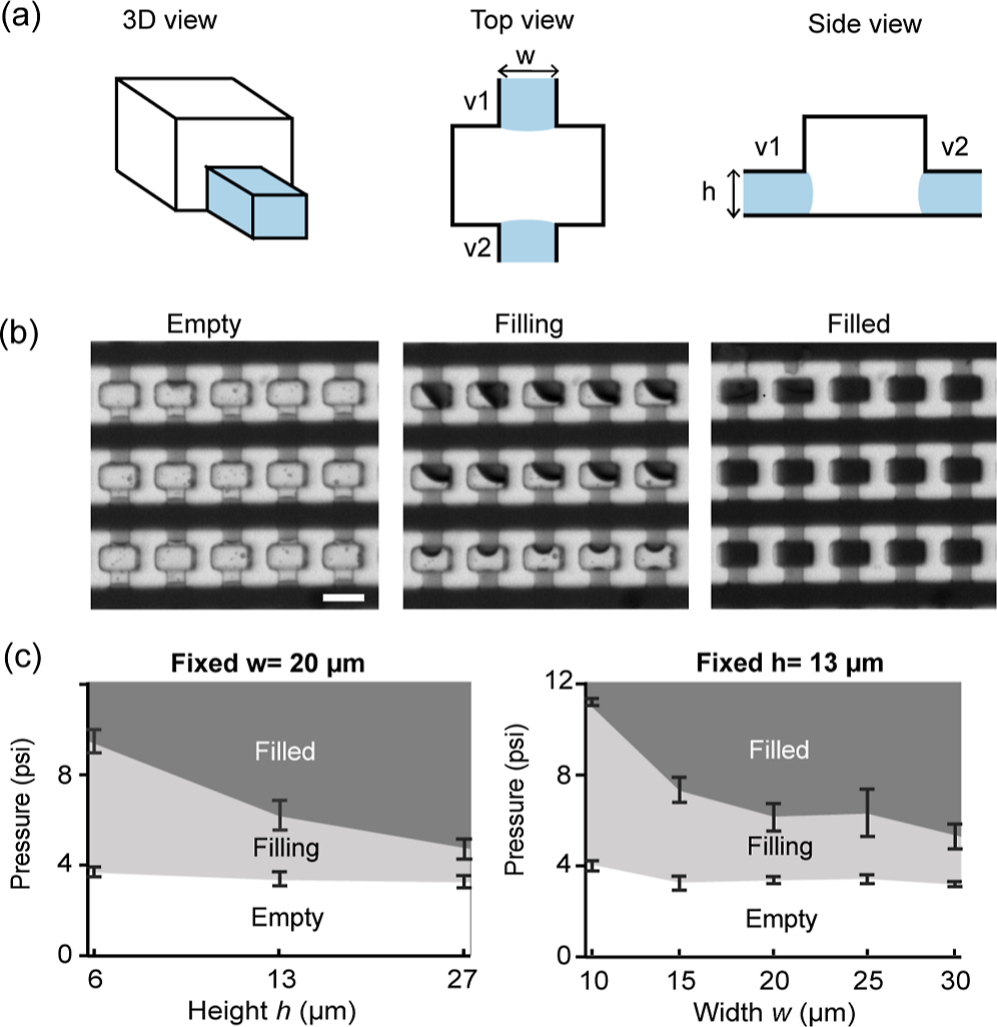
Characterization of the operation of the capillary valve network. (a) Capillary stop valve design relative to chamber dimensions. Stop valves are present on both sides of the chamber. (b) Three regimes of valve operation: first the chambers are empty with channel only filling (P_l_ < P_b_), then the chamber starts filling (P_l_ > P_b_), and finally the chambers are completely filled (no remaining air). Scale bar is 60 μm. (c) Valve filling regimes as a function of command pressure and valve geometry. One dimension changed while the other dimension held constant. Three devices were tested for each geometry. Error bars are device-to-device standard deviation.

We screened different driving pressures for each array geometry and tracked the filling state. We identified three regimes named “empty”, “filling”, and “filled” (Figure 2b). The “empty” regime is the desired regime in which the fluid stops before entering the chamber. In the “filling” regime, the valves break and the liquid starts entering the chambers. In the “filled” regime, the liquid completely fills the chambers. Both “filling” and “filled” regimes are failing cases.

As Figure 2c shows, a reliable valve operation range for targeted wetting is possible for all designs that we tested. The boundary between empty and filling regimes remains within 3.5–4 psi across the width and height combinations tested. In contrast, the boundary between filling and filled regimes shows a larger dependence on the valve dimensions. From Figure 2c, one can extract practical guidelines for a selective surface coating protocol. For the initial filling of the array, staying below 4 psi should ensure the device remains in the empty regime. Then, after the washing step, working at or above 12 psi will ensure the complete and quick filling of the chambers. These results highlight working ranges that are broad and easily achievable. It is therefore possible to deliver the different pressure commands without a precise pressure controller, facilitating device operation in a biosafety cabinet, which is a sterile environment with limited space for external equipment. Also, during our assays, we noted that it took only a few seconds to fill in the array, which makes the procedure very efficient (Movie S1). Finally, if one seeks to coat a larger surface, two strategies can be implemented. First, one can increase the array size. We have demonstrated similar results with arrays twice as large. Second, one can multiply the designs and connect them in parallel. Such arrangement allows for scaling up the coating while using a unique inlet pressure. Note that the driving pressure remains the same for one or multiple arrays in this configuration, ensuring proper liquid filling of the serpentine but not the chambers. The filling duration remains very short, less than 10 s (Movie S2). Note that the operating pressure is chosen to be well below the minimum burst pressure across the array, thus ensuring robustness despite pressure decay along the channel. Altogether, these results demonstrate that the method is scalable.

### Stable Capillary Valves Enable Protein Surface Coating Protocols

In protein patterning and cell assay applications, surface coating takes anywhere from one to several hours for sufficient molecular adsorption and complete surface coverage.^38,39^ Therefore, applying our method to protein patterning requires the targeted wetting to be stable for several hours. One challenge is that evaporation and absorption of water in the PDMS may affect the surface properties over time and degrade the capillary valve performances. The capillary valve performances may further change depending on the nature of proteins and other molecules in the buffer or medium that can act as surfactants and, therefore, alter the interfacial tension and contact angle. In particular, anionic surfactants near their critical micelle concentrations (CMCs) tend to enhance corner flow due to the Marangoni effect in liquid films.^42^ Therefore, the presence of a surfactant may decrease the capillary valve burst pressure and disrupt targeted liquid transport.

To determine the impact of these factors, we assessed the stability of the selected liquid filling in the capillary valve arrays over time. We tested a 4% BSA solution and a 1% Pluronic solution, as an example of protein and synthetic polymer, respectively; both are commonly used as surface blocking agents in biological applications. Both compounds lower the surface tension of water: BSA is an anionic surfactant, whereas Pluronic is a nonionic one. BSA and Pluronic were used at high concentrations to ensure complete surface coverage and thus far above their respective CMCs. We also tested pure DI water as a control, i.e., without surfactant. We injected the liquids in the serpentine channels at 1 psi and then incubated for 24 h as an estimate of an upper limit of the incubation time needed for achieving complete surface coverage.

We characterized the fraction of valves that stopped the liquid initially and 24 h after introduction of the liquid (Figure 3). Figure 3a shows images of the same array at the beginning and end of the assay. For visualization purposes, a food dye solution was used here but no food dye was introduced in the characterization experiments. One can clearly visualize that selected filling remains stable in most of the array through 24 h. Figure 3b quantifies the fraction of functional valves at both time points. Overall, the fraction of functional valves across all conditions decreases over the 24 h period but remains above 90%. We attribute the small fraction of defective valves to microfabrication defaults that impact the channel geometry and result in slowly leaking stop valves. Robust selective filling demonstrated here is important as it shows that both BSA and Pluronic solutions remain in the desired “empty” regime during the entire incubation time. This period is long enough to allow molecules to adsorb onto and fully cover the serpentine channel surface. Therefore, BSA and Pluronic solutions can be used for blocking the capillary network.

**Figure 3:**
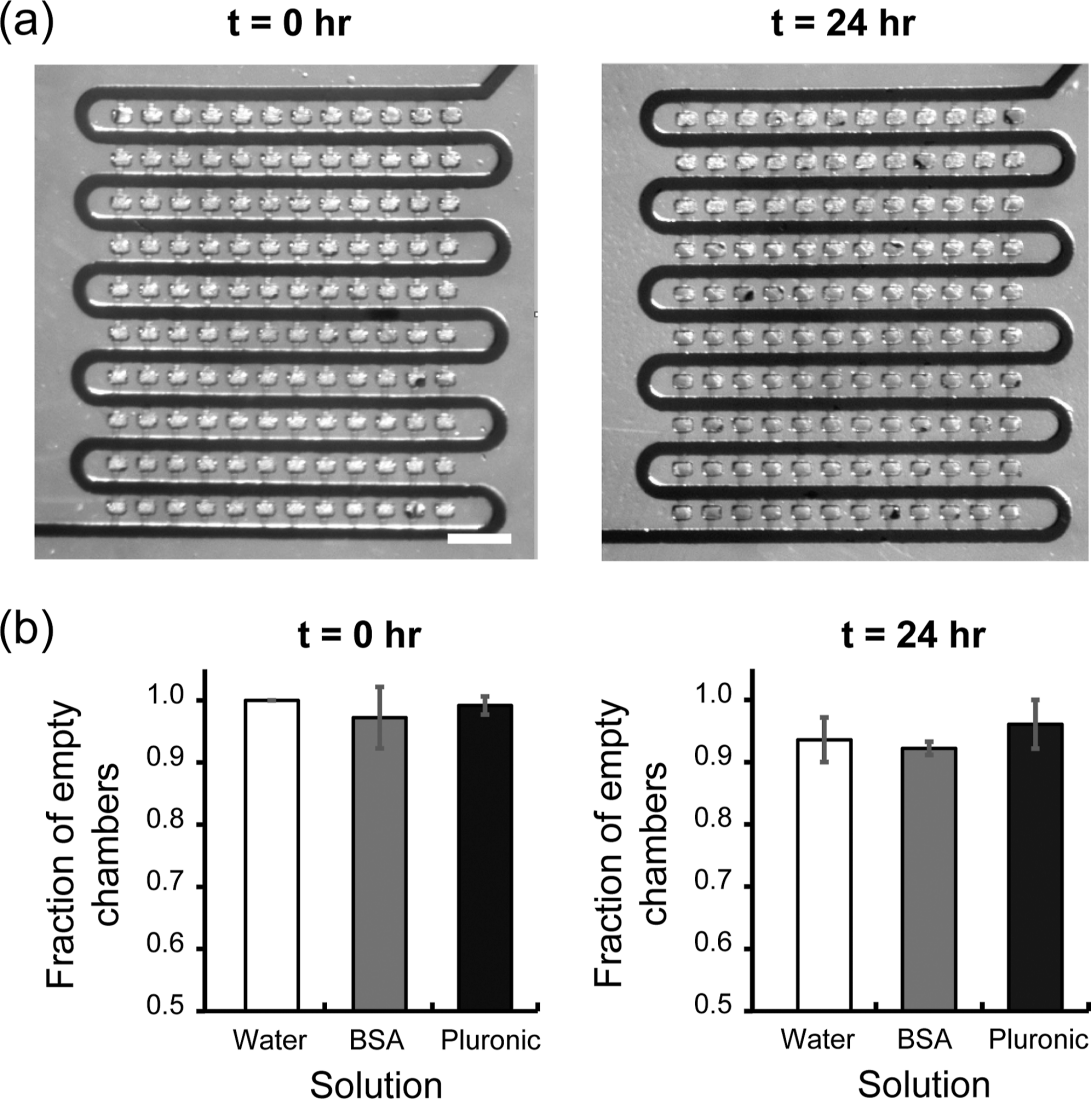
Robustness of the capillary valve network over 24 h. (a) Representative images of food dye solution initially filling channels (0 h) and remaining in channels after 24 h of incubation. Food dye solution was injected into the device at 1 psi. Scale bar is 200 μm. (b) Fraction of empty chambers in each device when filled with different solutions at 0 and 24 h. Three arrays were tested for each condition. Error bars are array-to-array standard deviation.

### Demonstration of 2D Patterning with Precise Pattern Registration

Sequential operation of the capillary stop valves allows selective patterning of the device surface with different reagents. Following surface treatment by the first reagent in the serpentine channel and a wash step, the capillary valves are broken, such that a second reagent can be introduced into the chambers. To use such patterning in cell-based assays, we need precise and sustained patterning over the course of several hours. Protein absorption stability on the PDMS surface has been well studied,^43–45^ determining in particular that fibronectin retains its bioactivity and can promote cell culture over multiple days. However, potential challenges in pattern registration may arise for two reasons. First, insufficient washing can cause some molecules of the first compound to deposit in the untreated chambers. Second, incomplete surface coverage by the first compound can result in some molecules of the second compound absorbing the serpentine channel. Additionally, surface diffusion of any remaining unbound molecules is a potential source of contamination of the pattern over time.

We assessed the quality of our method by patterning BSA molecules conjugated with two different fluorophores, fluorescein isothiocyanate (FITC) and TXR. We injected and incubated 1% BSA-FITC solution in the serpentine channel for 12 h, then washed and degassed the device with PBS. We then filled the chambers with 0.1% BSA-TXR solution, and upon 2 h of incubation, washed the devices again with PBS prior to imaging. The fluorophore-tagged proteins and concentrations used here were chosen for visualization purposes and not for cell patterning.

Fluorescent images of the patterned surface show uniform antibody staining with distinct menisci from pinning at the capillary valves (Figure 4a). The merged composite image also highlights that there is minimal overlap between the two surface treatments. A merged composite image of an entire patterned array is shown in Figure S1. To assess the accuracy of spatial registration, we quantified the fluorescence intensity line plots along each capillary valve (Figure 4b). The results show sharp changes in intensity on either side of the valves. The green channel intensity shows a sharp decay from the serpentine channel to the chamber, indicating that the serpentine channel is coated with BSA-FITC. The red channel intensity shows a sharp increase from the serpentine channel to the chamber, indicating that the chamber is coated with BSA-TXR. The very sharp spikes in intensity are located at the device walls where BSA is coated vertically, resulting in much larger signals than just from the coating on the top and bottom surfaces of the device. The transition in the fluorescence signal is less than 10 μm on both sides of the 40 μm wide chamber. This resolution is enough for the target application of culturing adherent cells. Should there be applications requiring smaller patterns, the standard soft lithography process can easily achieve channels of 5 μm. In addition, we characterized the uniformity of the patterning for each row of an array (Figure S2) and show variations in the order of 10% or less from one row to the next. These data validate the method for its ability to register 2D patterns with high precision and negligible cross contamination.

**Figure 4:**
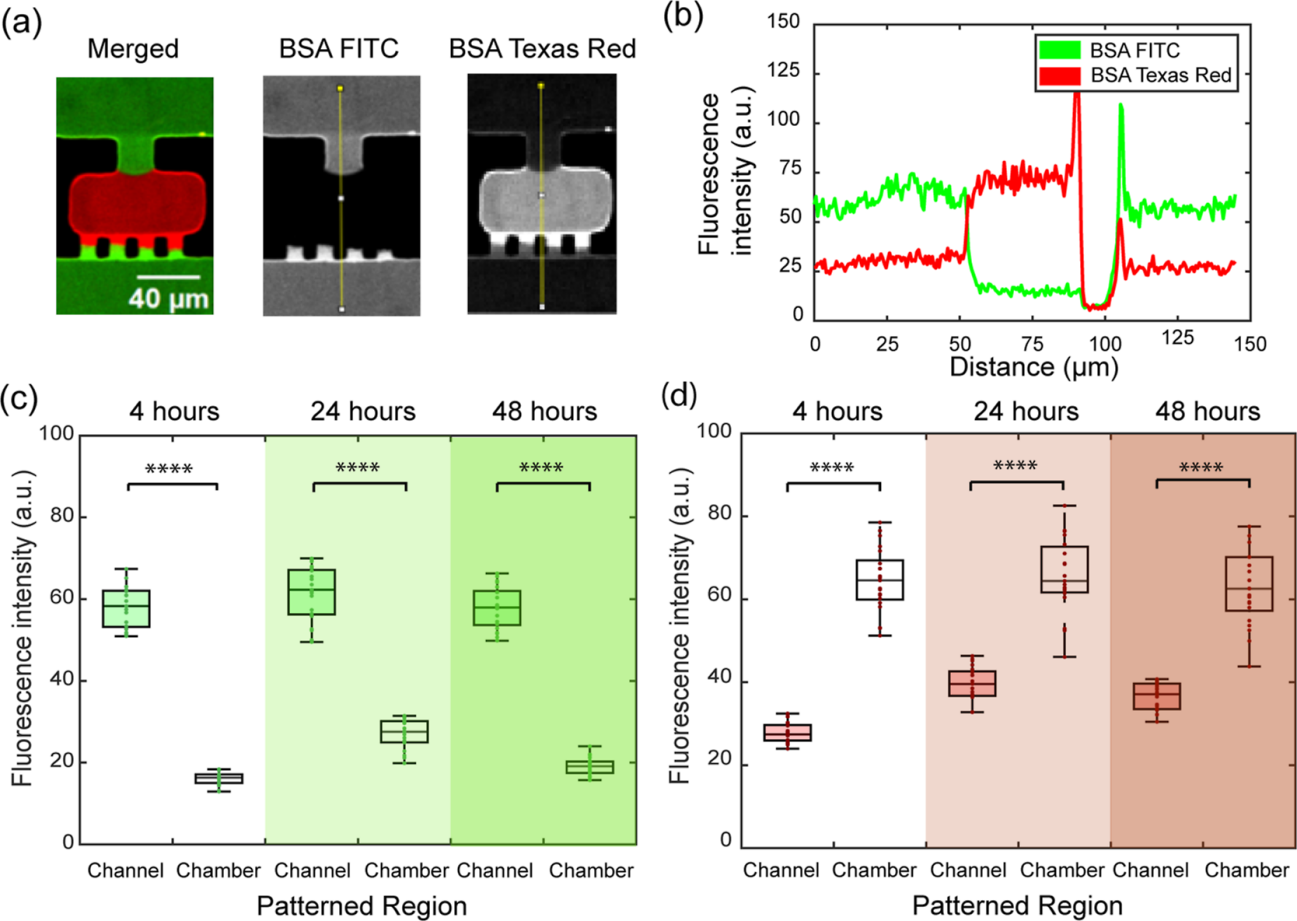
Characterization of selective surface coating. (a) Contrast adjusted fluorescence images for BSA-FITC (green) and BSA-TXR (red); (b) intensity distributions of the two fluorescent proteins across the chamber; (c) fluorescence intensity of BSA-FITC in a patterned array in the serpentine channel and chambers at three time points; (d) same for BSA-TXR. Channel and chamber locations were randomly sampled in the capillary valve array. **** denotes *p* < 0.0001, by Student’s *t*-test.

To determine the robustness of the patterning, we sampled the fluorescence intensity across various locations of the array and different time points. Figure 4c,d shows the quantification for the BSA-FITC signal and BSA-TXR signal, respectively, at 4, 24, and 48 h after patterning the second protein. The contrast between BSA-FITC in the channel (upstream of the capillary valve) and in the chamber (downstream of the capillary valve) is relatively stable over time, as is the contrast between BSA-TXR in the channel and the chamber. These data suggest that our coating strategy can yield controlled surface density of the coated proteins that remains stable over the course of a multiday assay on-chip.

### Selective Patterning of On-Chip Cell Culture

To demonstrate the efficacy of selective protein patterning in guiding selective cell growth, we applied our protocol to create an adherent surface coating in the chambers only and performed cell culture in the patterned device. We needed to confirm that selective blocking prevented cell adherence in the channels but did not prevent cell adherence to the chambers.

In the selective surface coating condition (SC), we used a 4% BSA solution and a 1 μg/mL fibronectin (FN) solution to pattern the serpentine channel and chambers, respectively. Control devices were coated with either 1 μg/mL FN everywhere or 4% BSA everywhere. We used an epithelial cell line (HT-1080 cells) and loaded the devices with 50 μL of a cell suspension at a density of 0.5 million cells/mL. Following cell loading into the array chambers, we supplemented the devices with continuous perfusion of cell media to support cell growth. The protein concentrations used in this study were optimal for the HT-1080 cells but may need to be adjusted for other cell lines.

Figure 5a shows representative images of cells in the three experimental conditions upon cell loading (*t* = 0) and after 20 h of on-chip culture (see Figure S3 for pictures of the whole field of view at *t* = 20 h). Two devices were tested per condition. Each device contains four arrays; this represents 672 chambers per device. Cell loading successfully led to homogeneous cell distribution across the arrays (Figure S4). At *t* = 0 h, cells in all conditions look similar: cells are in suspension and trapped in the chambers due to hydrodynamic flow focusing. At *t* = 20 h, the cells show differences in localization. One can see many cells in the serpentine channel for the condition with FN everywhere. In contrast, we observe a fewer number of cells in the channel for the selective coating and BSA-everywhere conditions. Live–dead cell staining at 20 h established that over 80% of cells were alive in all conditions (Figure S5). We note that there are some cells remaining in the channels and these aggregates may restrict flow in some of the arrays. To prevent this, future design improvements should include widening the main channel and adjusting the width of the chambers proportionally for cell loading.

**Figure 5:**
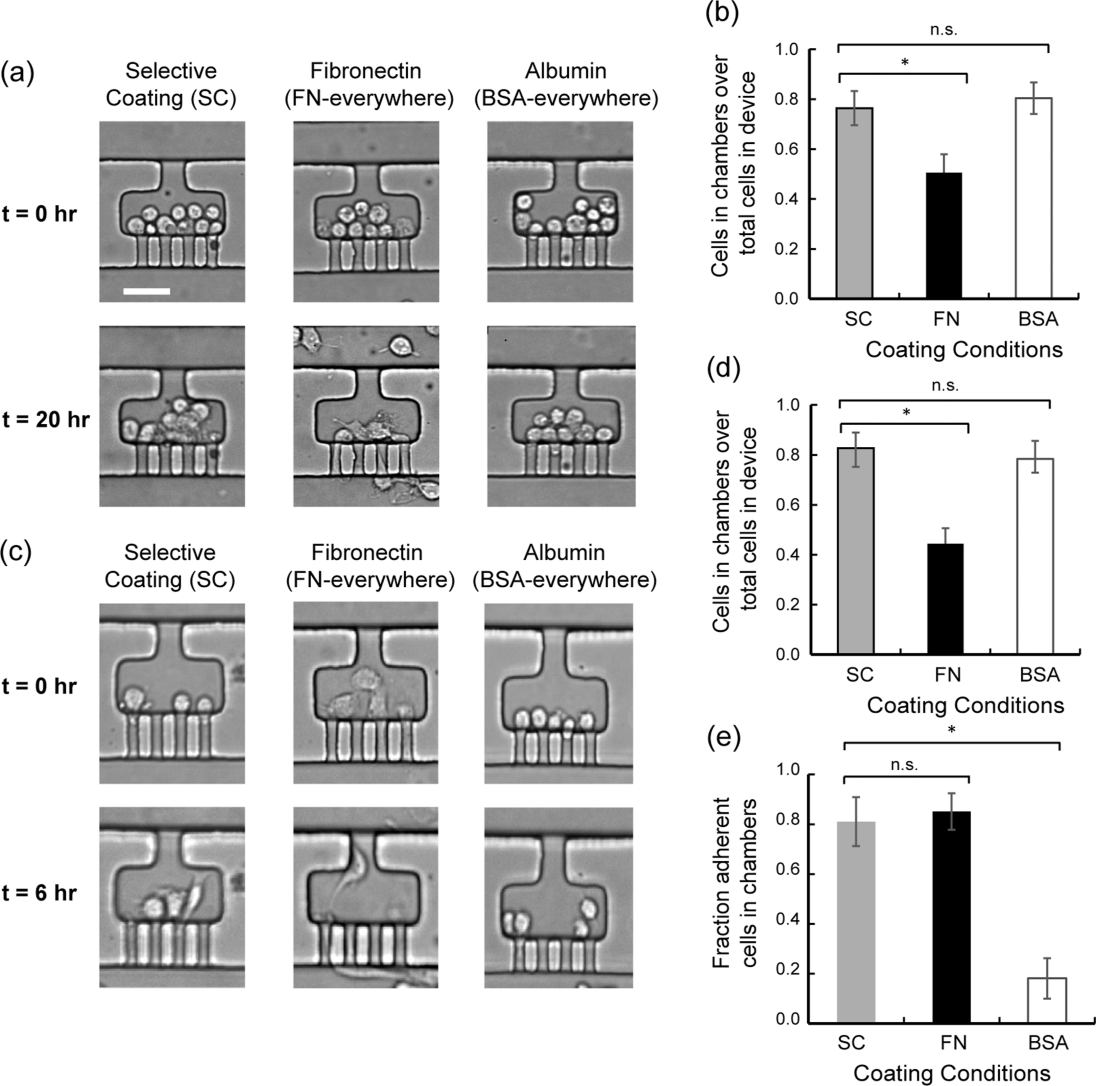
On-chip culture of adherent cells. (a) Representative images of HT-1080 cells cultured in devices with different surface treatment, after cell loading and 20 h after on-chip culture. Media perfusion is from the top of the image. Scale bar is 40 μm. (b) Fraction of HT-1080 cells in chambers compared to total cell count in arrays 20 h after cell loading. A higher fraction is representative of better cell retention. (c) Representative images of 3T3 cells cultured in devices with different surface treatment, after cell loading and 6 h after on-chip culture. Media perfusion is from the top of the image. Scale bar is 40 μm. (d) Fraction of 3T3 cells in chambers compared to total cell count in arrays 6 h after cell loading. (e) Fraction of HT-1080 cells remaining in chambers after exposure to reverse flow for 1 min. A high fraction is indicative of greater cell adherence. One star indicates a *p*-value < 0.05; two stars indicate a *p*-value < 0.01 (by Student’s *t*-test). Error bars are array-to-array standard deviation across 8 arrays.

To assess cell retention, we quantified the percentage of cells in the chambers relative to the total cell count in each array after 20 h of on-chip culture (Figure 5b). BSA blocking was successful as the SC condition shows a higher fraction of cells remaining in the chambers than the FN-everywhere condition at 20 h. The BSA-everywhere condition shows a fraction of cells remaining in the chambers equivalent to the SC condition. This result is not surprising as one expects that the cells cannot adhere anywhere in the BSA-coated device. Therefore, the cells remain in suspension and are trapped due to the same hydrodynamic flow focusing mechanism as for loading.

We also tested our selective coating technique with another cell line (NIH 3T3). The SC condition for 3T3 cells shows the preferred location of the cells within the chambers (Figure 5c), demonstrating the generalizability of our method. We also characterized cell retention at 6 h (Figure 5d). The 3T3 cells are very motile; cell retention may be limited by BSA surface complex formation and desorption kinetics.^46^ For a longer lasting surface pattern, covalent surface functionalization^47^ may be used in combination of our selective surface patterning protocol.

To confirm cell adherence or the lack of in the different surface coating conditions, we tested cell adherence for the HT-1080 cells after 20 h of culture by reversing the flow of cell media in the devices. By doing so, the nonadherent cells should be pushed out of the chambers and be removed by the flow in the serpentine channel. We quantified the fraction of cell remaining in the chamber after 1 min of reverse flow at 1 psi, which corresponds to the fraction of adherent cells in the chambers (Figure 5e). In the BSA-everywhere condition, over 80% of cells get pushed out of the chambers and into the serpentine channel immediately after back pressure is applied. In contrast, under the selective coating and FN-everywhere conditions, nearly 80% of cells remain in the chambers. Furthermore, we note that chamber size does not impact cell adhesion (Figure S6) and that adhesion is uniform across the whole array (Figure S7). Cell adhesion variability is comparable to the variability in cell loading (Figure S4), with minimal row-to-row variation. These results demonstrate that selective coating successfully attains both cell confinement and adherence during on-chip culture.

## Conclusions

We have demonstrated a method for selective surface coating that allows for the generation of 2D patterns in hard-to-access surfaces such as closed microfluidic devices. Using only the device geometry, pressure-driven flow, and sequential reagent delivery, one spatially and temporally controls the exposure of the channel surface to blocking agents and cell adhesion molecules separately. With this technique, we demonstrated 2D patterning of hundreds of spots and performed on-chip culture over 20 h. The method is simple, cost-efficient, and scalable and can be performed on a variety of substrates, which opens a broad range of applications. It is simple because little manual intervention is necessary and the large range of operating conditions makes the process user-friendly. The method is cost-efficient because it uses only capillary forces already realized by the design geometry; no expensive equipment or material is necessary. The method is scalable because the patterning protocol can be performed in parallel for many devices. Importantly, this method can be used after microfluidic channels are sealed, avoiding harsh bonding processes that could damage the coating materials, e.g., altering the adherent proteins’ activity.

This method may be of interest for a variety of applications. We demonstrated 2D patterning for adherent cell culture; such cell arrays can be used for tissue-on-chip and organ-on-chip applications, as well. In addition, our selective surface coating technique may be used for arrays of other entities, such as bead, spheroids, or organoids.^48,49^ Scaling up the device dimensions does not impact the capillary valve operation. Beyond cell applications, protein patterning is also commonly used for antibody assays for biomolecule detection.^50^ Finally, controlled surface properties may be of interest for varying wetting properties, which is important in droplet microfluidics for double emulsion. Therefore, we envision this method to be of use for a broad range of applications involving surface-based processes.

## Supplementary Material







## Notes

Results: comparison of performances of techniques for coating closed microchannels; representative image showing selective coating in an array; characterization of coating uniformity; whole field of view images of the on-chip cell culture devices for the different coatings; characterization of cell distribution in the array; cell viability results; impact of chamber size on cell adherence; and characterization of cell adhesion uniformity across the array (PDF)
Results: different filling regimes of the microfluidic chambers based on input pressure (MP4)
Successful filling of the serpentine channel but not the chambers for larger arrays (MP4)
