# Supplementary material for: Self-Regulated Selective Surface Coating Enables Confinement of Adherent Cells in Closed Microfluidic Arrays
Source: Anal Chem. 2025 Nov 17;97(47):25958–66. doi: 10.1021/acs.analchem.5c02622 (PMC12676511; doi:10.1021/acs.analchem.5c02622)
Supplement: Supplementary file 1 [file ac5c02622_si_001.pdf]

Supporting Information for

Self-Regulated Selective Surface Coating Enables Confinement of Adherent Cells in Closed Microfluidic Arrays

Anna Kaehr,†[a] Guillaume Aubry,†[a] and Hang Lu [ab] *

[a] School of Chemical & Biomolecular Engineering, Georgia Institute of Technology, 311 Ferst Drive NW, Atlanta, Georgia 30332, USA.
[b] Interdisciplinary Program in Bioengineering, Georgia Institute of Technology, 311 Ferst Drive NW, Atlanta, Georgia 30332, USA.

Corresponding Author

* Hang Lu: hang.lu@gatech.edu

Author Contributions

† These authors contributed equally to this work.

Table of Contents

Table S1: Comparison of performances of methods capable of patterning closed microfluidic channels p.S2

Figure S1: Representative image of the selective surface coating of a whole array . p.S3

Figure S2: Characterization of the uniformity of the selective surface coating across the array p.S4

Figure S3: Representative images of whole arrays showing cell cultures under different conditions after t = 20 hrs p.S5

Figure S4: Characterization of cell distribution upon loading across the array …….. p.S6

Figure S5: HT-1080 cell viability 20 hours after cell loading p.S7

Figure S6: Characterization of cell adherence in chambers of different sizes ……... p.S8

Figure S7: Characterization of cell adhesion uniformity across the array ………….. p.S9

Movie S1: Different filling regimes of the microfluidic chambers based on input pressure (.mp4)

Movie S2: Successful filling of serpentine channel but not the chambers for larger arrays (.mp4)

**Technique**	**Photopatterning [1]**	**Laminar flow patterning [2]**	**Capillary flow patterning [3,4]**	**Our method**
Reliability	Very (robust control of illumination pattern)	Moderate (depends on flow stability)	Very (flow robustly controlled using capillary flow)	**Very reliable** (flow robustly controlled using capillary valves)
Throughput: (1) patterning speed, (2) number of set ups easy to implement	Low: (1) ~ 2 mm^2/hr & (2) x1 set-up because of costs	Moderate: (1) ~ 1 mm^2/hr & (2) x5 set-ups (using multichannel syringe pumps)	High: (1) ~ 10 mm^2/hr & (2) x10 set-ups (no peripheral equipment)	**High throughput:** (1) ~ 10 mm^2/hr & (2) x10 set-ups (no peripheral equipment)
Cost	High: ~$200000 (Confocal microscope)	Moderate ~ $500-1000 for two syringe pumps	Very low: ~$10 (no peripheral equipment)	**Very low cost**: ~$10 (no peripheral equipment)
Protocol accessibility	Difficult: Protocol requires confocal microscope	Easy: only syringe pumps required, easy to operate	Very easy: no expertise and no equipment required	**Very accessible**: no expertise and no equipment required
Resolution	~ 1 µm (optical diffraction)	~ 5 µm (flow mechanics)	~ 5 µm (channel dimension)	**~ 5 µm** (channel dimension)
Resolution demonstrated	5 µm	10 µm	5 µm	40 µm
Achievable shapes: Continuous stripes	yes	yes	yes	**yes, can generate continuous stripes**
Achievable shapes: spots	yes	no	no	**yes, can generate spots**

[1] Kling, A., et al, Laser-assisted protein micropatterning in a thermoplastic device for multiplexed prostate cancer biomarker detection. Lab Chip, 2023. 23(3): p. 534-541.
[2] Takayama, S., et al., Patterning cells and their environments using multiple laminar fluid flows in capillary networks. PNAS, 1999. 96(10): p. 5545-5548.
[3] Lee, S.H., et al., Capillary Based Patterning of Cellular Communities in Laterally Open Channels. Analytical Chemistry, 2010. 82(7): p. 2900-2906.
[4] Papadimitriou, V.A., et al., 3D capillary stop valves for versatile patterning inside microfluidic chips. Analytica Chimica Acta, 2018. 1000: p. 232-238.

Table S1. Comparison of performances of methods capable of patterning closed microfluidic channels

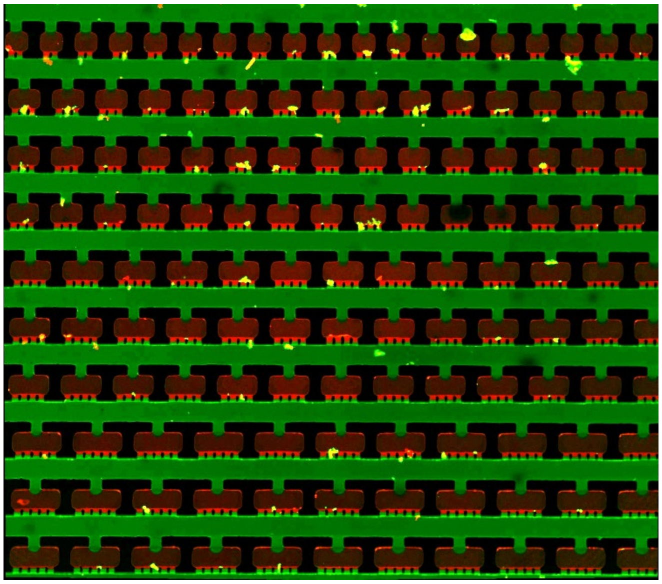

Figure S1. Representative image of a whole array (rows 3-12) showing selective surface coating across the array. The image is contrast adjusted, with BSA-FITC shown in green and coating the serpentine channel and BSA-TXR shown in red and coating the chambers.

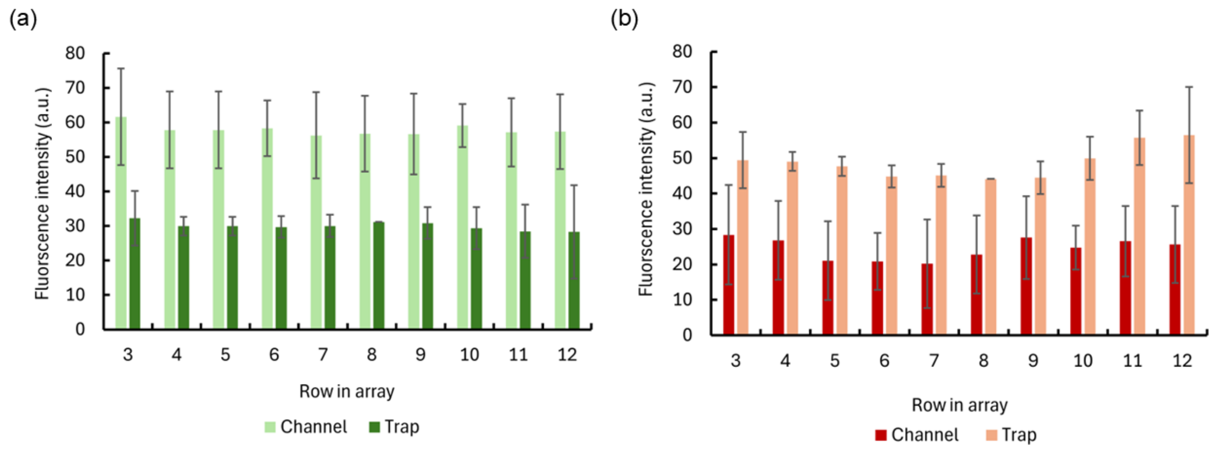

Figure S2. Characterization of the uniformity of the selective surface coating across the array. (a) Fluorescence intensity of BSA-FITC quantified for each row of the array in the serpentine channel (light green) and chambers (dark green). (b) Fluorescence intensity of BSA-TXR quantified for each row of the array in the serpentine channel (dark red) and chambers (light red). While there are 12 rows in the array, the first 2 rows are left out due to the presence of debris in the chambers. Error bars are the standard deviation of intensity values across 14 traps in a row.

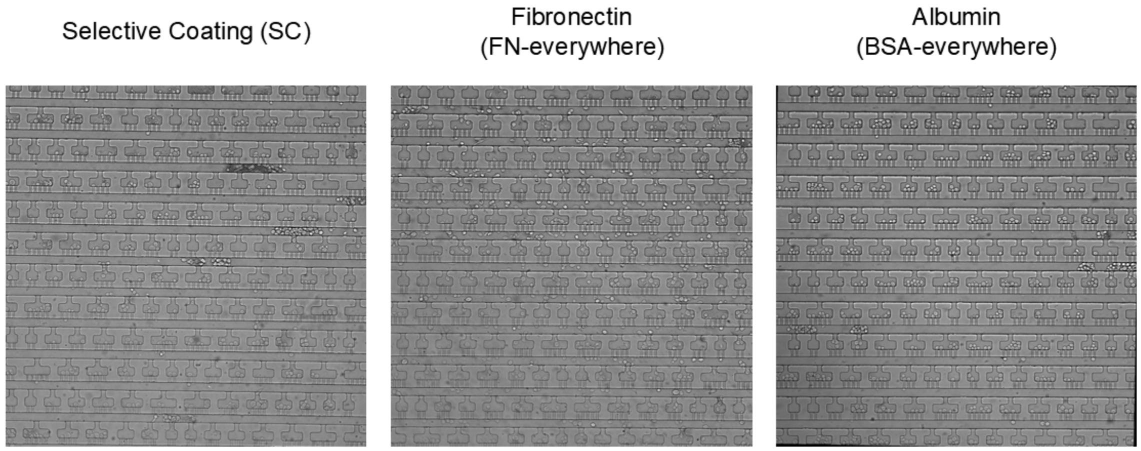

Figure S3. Representative images of whole arrays showing HT-1080 cell cultures under different conditions after t = 20 hrs. “FN-everywhere”, “SC condition”, and “BSA-everywhere” indicate cell culture on chips coated with fibronectin everywhere, fibronectin the chambers only, and BSA everywhere respectively.

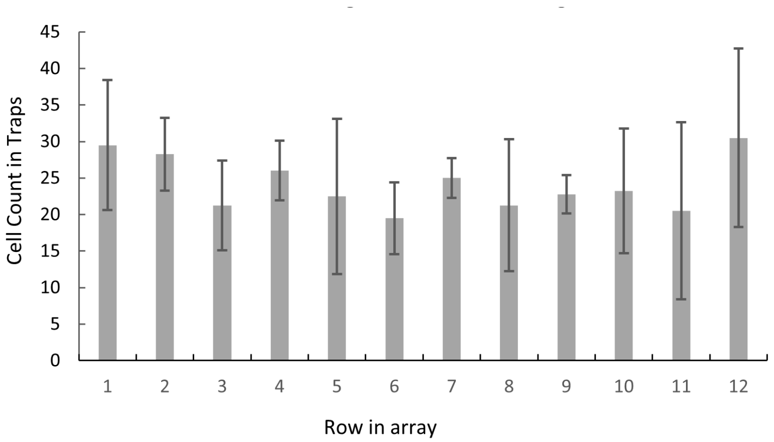

Figure S4. Characterization of cell distribution upon loading across the array. Cell count is an average across n=4 arrays. There are 12 rows per array and 14 chambers per row. Error bars are standard deviations of the number of cells in a row between the different arrays. Cell loading is independent from the surface coating condition. Cell loading is entirely determined by the array geometry.

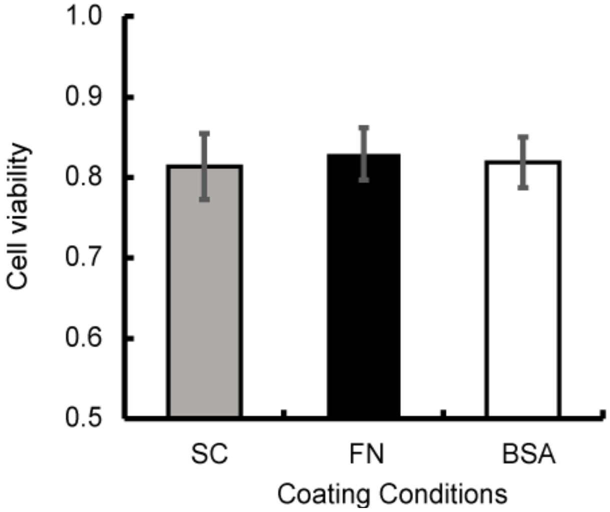

Figure S5. Cell viability 20 hours after cell loading. A live-dead assay was performed using Calcein AM and Ethidium Homodimer. Error bars are array-to-array standard deviation.

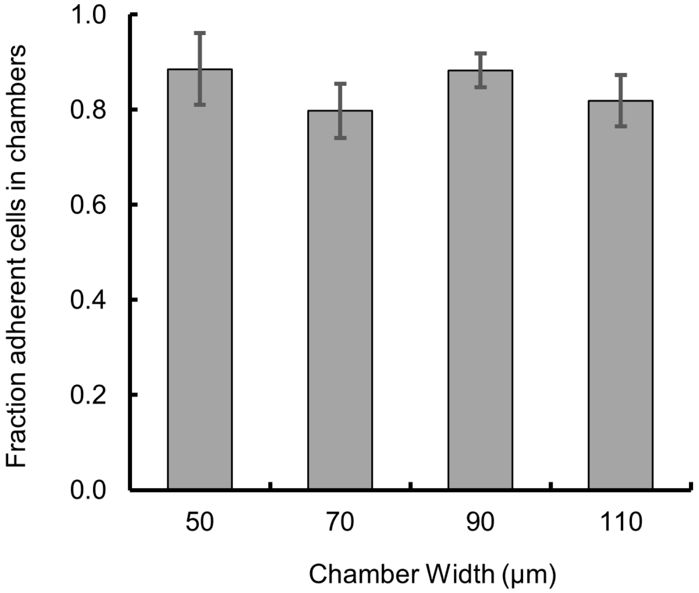

Figure S6. Characterization of cell adherence in chambers of different sizes. While the larger chambers load more cells, there is no significant difference in the fraction of adherent cells to total loaded cells across chamber types. Cell adherence is quantified via flow-reversal test.

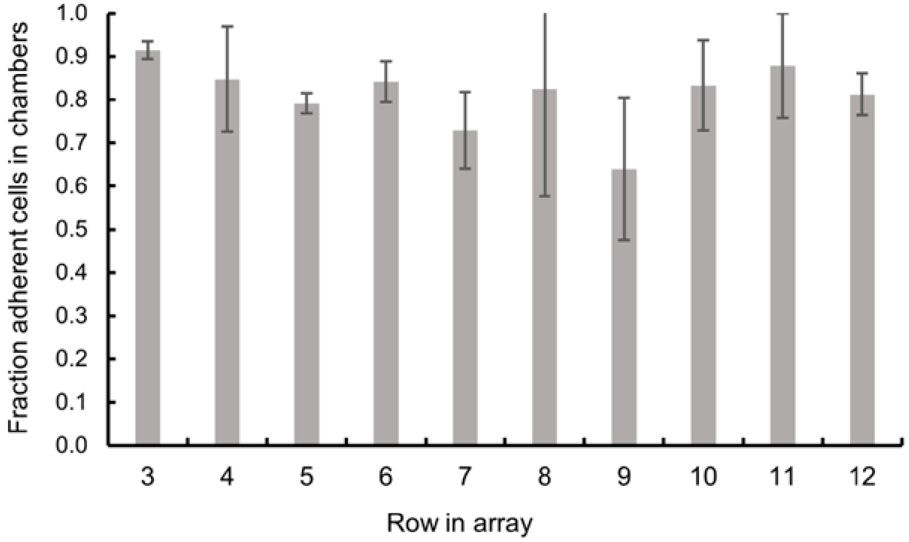

Figure S7. Characterization of cell adhesion across the array after 20 hours of HT-1080 cell culture in devices with selective surface coating. Cell adherence is independent from the surface coating condition and shows insignificant differences across the array. Cell adherence is an average across 14 chambers per row in n=3 arrays. While there are 12 rows in the array, the first 2 rows are left out due to the presence of debris in the chambers.
